# HIV and Hodgkin Lymphoma Survival: A Prospective Study in Botswana

**DOI:** 10.1200/GO.21.00163

**Published:** 2022-01-13

**Authors:** Kaelo Moahi, Tlotlo Ralefala, Isaac Nkele, Scott Triedman, Aliyah Sohani, Zola Musimar, Jason Efstathiou, Philipe Armand, Shahin Lockman, Scott Dryden-Peterson

**Affiliations:** ^1^ Department of Medicine, Hospital of the University of Pennsylvania, Philadelphia, PA; ^2^ Department of Pediatrics, Children's Hospital of Philadelphia, Philadelphia, PA; ^3^ Princess Marina Hospital, Ministry of Health and Wellness, Gaborone, Botswana; ^4^ Botswana-Harvard AIDS Institute Partnership, Gaborone, Botswana; ^5^ Department of Radiation Oncology, Warren Alpert Medical School, Providence, RI; ^6^ Department of Pathology, Massachusetts General Hospital, Boston, MA; ^7^ Department of Pathology, Harvard Medical School, Boston, MA; ^8^ Department of Radiation Oncology, Massachusetts General Hospital, Boston, MA; ^9^ Department of Medical Oncology/Hematologic Malignancies, Dana-Farber Cancer Institute, Boston, MA; ^10^ Department of Immunology and Infectious Diseases, Harvard T.H. Chan School of Public Health, Boston, MA; ^11^ Division of Infectious Diseases, Brigham and Women's Hospital, Boston, MA

## Abstract

People living with HIV (PLWH) experience increased risk of Hodgkin lymphoma (HL) despite effective initiation of antiretroviral therapy (ART). In high-income countries, outcomes following HIV HL have been reported to be non-differential, or inferior for PLWH. We sought to assess the effect of HIV on HL survival in Botswana, an African country with a generalized HIV epidemic and high ART coverage, to describe a context more reflective of global HIV populations.

**PATIENTS AND METHODS:**

In the Thabatse Cancer Cohort, consenting participants initiating treatment for HL at one of four cancer centers in Botswana were enrolled from 2010 to 2020. Patients were followed quarterly for up to 5 years. The impact of HIV on survival following treatment initiation was assessed using an inverse probability–weighted Cox marginal structural model adjusted for age, performance status, and disease stage.

**RESULTS:**

Seventy-eight new HL cases were enrolled, 47 (60%) were PLWH and 31 (40%) were HIV-uninfected. Baseline characteristics were similar between groups. The majority (61%) of patients presented with regional disease (stage I or II) with no statistically significant difference by HIV status (*P* = .38). Nearly all (87%) PLWH participants were on ART before their HL diagnosis (median ART duration 42 months), and median CD4 count was 413 cells/μL (interquartile range 253-691). Survival, in unadjusted analyses, was lower among patients without HIV compared with PLWH (log rank *P* = .021). In adjusted analysis, HIV infection was not significantly associated with survival in inverse probability–weighted Cox model (hazard ratio 0.43; 95% CI, 0.16 to 1.16; *P* = .094).

**CONCLUSION:**

In this cohort of patients treated for HL in Botswana, survival in PLWH (87% on long-standing ART) was at least as good as in individuals without HIV.

## INTRODUCTION

Low- and middle-income countries with high HIV prevalence are especially affected by the global rise in cancer incidence.^1,2^ The proportion of people living with HIV (PLWH) who are receiving antiretroviral therapy (ART) in Botswana is among the highest in the world, but PLWH are nevertheless contracting cancer at higher rates than their HIV-negative counterparts.^3-6^ PLWH are 3- to 10-fold more likely to develop Hodgkin lymphoma (HL) than HIV-uninfected populations. For those receiving ART, this risk swells to 20- to 30-fold.^7-10^ Previous studies suggest that as ART permits restoration of immune function in PLWH, increased leukocyte counts can support the tumor (Reed-Sternberg cells) microenvironment in ways that are less likely without ART.^11^ Indeed, HL in PLWH have been demonstrated to have unique gene expression and molecular patterns.^11-16^ Studies in Europe and the United States have looked to ascertain the presence of differential survival of HL, if any, between PLWH and their HIV-uninfected counterparts in the contemporary ART era.^17-19^ These have shown overall worse or nondifferent outcomes for PLWH.^17-19^ Such a study has yet to be conducted in the context of high adult HIV prevalence (> 20.3% in Botswana),^4^ in a resource-limited setting with high ART coverage.^14^ To describe experience in a context reflective of global HIV populations, we assessed the effect of HIV on HL survival in Botswana.

**CONTEXT**

**Key Objective**
Given the increased incidence of Hodgkin lymphoma (HL) in people living with HIV (PLWH) compared with those who do not have HIV, do PLWHIV with HL (HIV HL) have inferior mortality? We aimed to investigate this question in the context of a low- and middle-income country with a high HIV incidence, given this is the dominant HIV setting worldwide.
**Knowledge Generated**
Our findings suggest that in Botswana, mortality in HIV HL is noninferior to that of non-HIV HL in adjusted analysis. Our findings also suggest increased access to oncologic care for PLWH relative to those without HIV in Botswana.
**Relevance**
We should expect similar outcomes for those with HL irrespective of HIV status in Botswana's HL program in its current form, although this expectation may stem from the health system's inability to promptly treat malignancy in patients who do not regularly interface with it (those without HIV).

## PATIENTS AND METHODS

### Study Participants

As part of the Thabatse Cancer Cohort, Botswana citizens age 18 years or older receiving a new diagnosis of HL at one of the four principal cancer centers in Botswana (Princess Marina Hospital, Gaborone Private Hospital, and Bokamoso Private Hospital in Gaborone, and Nyangabgwe Referral Hospital in Francistown) were approached for enrollment. Patients unwilling or unable to provide informed consent or under involuntary incarceration were excluded. We used cases enrolled from October 2010 to January 2020. Written confirmation of informed consent was obtained from participants, and the study was approved by ethical review committees at Harvard T.H. Chan School of Public Health and Botswana Ministry of Health and Wellness.

Upon enrollment, we collected demographic information, disease presentation characteristics, and medical histories through interviews and reviews of medical records. Subjects without previously diagnosed HIV infection or without a negative HIV test within the prior 6 months were requested to test for HIV. The diagnosis of HL was made on the basis of surgical pathology reports generated at the Botswana National Health Laboratory. This included routine histology, as confirmatory immunohistochemistry or Epstein-Barr virus (EBV)-encoded RNA in situ hybridization was not available. Clinical examination, chest radiograph, and ultrasound were reliably used for staging. Cross-sectional imaging including computed tomography-positron emission tomography and bone marrow biopsy were not available to the majority of patients. When clinically noted, we abstracted presence of B symptoms. Patients were followed in person and by telephone quarterly through treatment and thereafter for up to 5 years. For participants who died during the study, cause of death was abstracted from an official death certificate when available. We were provided further information about deaths through discussions with patients' families and health care providers.

### Treatment

Botswana provides cancer and HIV treatment free of charge to citizens. No treatment guidelines were in place, and treatment approach varied by clinical scenario, hospital resources, and preference of the treating physician. In general, patients received four to six cycles of ABVD (doxorubicin, bleomycin, vinblastine, and dacarbazine). BEACOPP (bleomycin, etoposide, doxorubicin, cyclophosphamide, vincristine, procarbazine, and prednisone) was used as a second-line regimen for disease progression. Patients treated in the private sector frequently received Stanford V (doxorubicin, vinblastine, mechlorethamine, vincristine, bleomycin, etoposide, and prednisone). Adjuvant radiation was administered for those with persistent or residual disease after chemotherapy, or as consolidation for limited-stage disease. Autologous stem-cell transplantation was supported outside of Botswana for patients with relapsed disease. With the exception of radiation treatment, centralized records of administered treatment were not available. Consequently, treatment histories were obtained through review of medical records held by patients, sometimes by telephone for patients living remotely, and were not available for all patients.

International and Botswana HIV treatment guidelines evolved during the course of the study. Treatment was recommended for individuals with AIDS and those with CD4 cell count of 250 cells/μL or less before 2012, 350 cells/μL or less from 2012 to 2016, and then regardless of CD4 cell count (universal ART) in 2016. First-line ART regimens during the study included tenofovir and emtricitabine with combination with either efavirenz or dolutegravir; however, many patients were receiving historical regimens.

### Definitions and Data Analysis

Patients with documented HIV infection (record of positive serology, HIV RNA testing, or receiving ART) before or within 1 year following enrollment were considered HIV-infected.

Baseline characteristics were compared across HL patients with and without HIV using Fisher's exact and Wilcoxon rank-sum tests. Survival by HIV status was assessed with Kaplan-Meier method and log-rank test. With a limited number of events (deaths) and consequent increased risk of overfitting using standard approaches to multivariable regression, we used two analytic strategies. First, we used sequential bivariate Cox models to assess stability of HIV effect estimate with inclusion of sociodemographic factors (socioeconomic, education, wealth, income, smoking, and sex) and oncologic factors (treatment intent and treatment initiated). These factors did not modify the estimated effect of HIV infection by more than 25% and were not included in the final model. Subsequently, we built an inverse probability–weighted Cox model to assess the possible effect of HIV while adjusting for possible key factors. We limited factors in the logistic model calculating weights to three a priori selected predictors: age (greater or < 45 years), performance status (Eastern Cooperative Oncology Group 0 or 1 *v* 2, 3, or 4), and HL stage (I or II *v* III, IV, or unstaged).

## RESULTS

### Demographics

In total, 78 patients with HL were enrolled in the study between April 2011 and January 2020. Forty-seven (60%) were HIV-infected, whereas 31 (40%) were HIV-uninfected. In the larger cohort, approximately 5% of patients with cancer declined participation and 5% were too ill to be able to provide informed consent. We were unable to capture cancer type for nonparticipating patients. Median age was similar between PLWH and HIV-uninfected participants (40.7 and 31.8 years, respectively). Similar proportions of persons with and without HIV had received primary-level-only education; 30% of PLWH had personal income under $50 US dollars/month, whereas 68% of HIV-uninfected patients had monthly personal income under $50 US dollars/month (*P* = .001; Table 1).

**TABLE 1:**
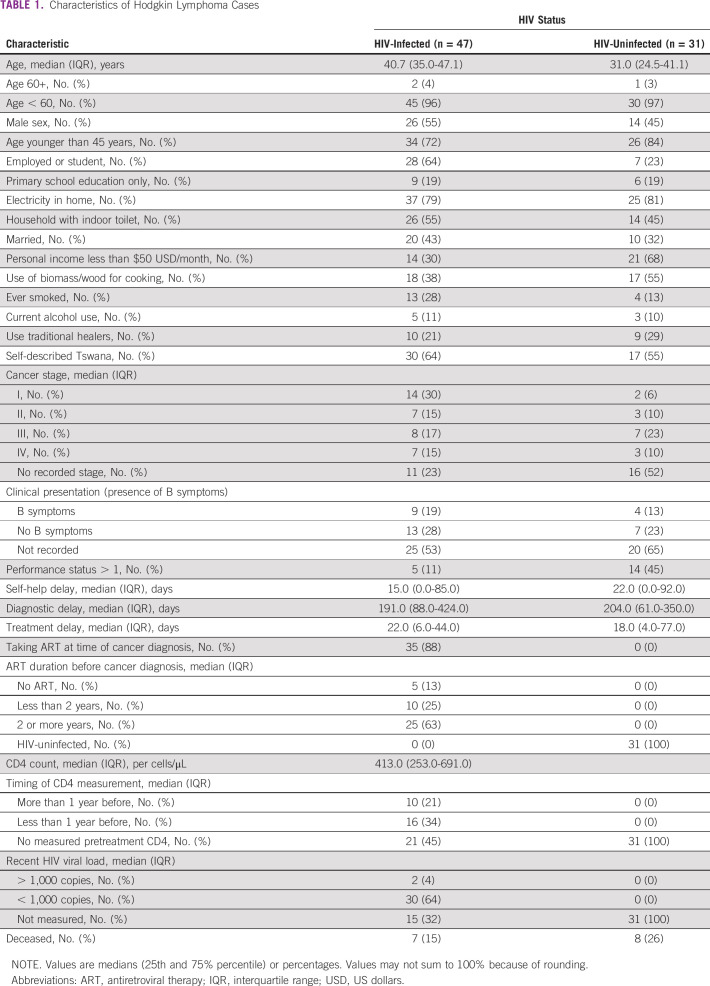
Characteristics of Hodgkin Lymphoma Cases

### Patient Presentation

Thirty-two (32%) of PLWH presented with late-stage disease (III or IV), whereas 10 (33%) of their HIV-uninfected counterparts presented with late-stage disease (*P* = .26). Nearly all (88%) of PLWH were on ART before their HL diagnosis. Median enrollment CD4 count for PLWH was 413 cells/μL (interquartile range [IQR] 253-691) upon study enrollment. HL subtype on pathologic reports was included for 52 patients, with subtype data missing for the remaining 26 (Table 2). Participants self-reported time between initial symptoms and care seeking. This interval was shorter in PLWH (15.0 days, IQR 0.0-85.0 days) than in their HIV-uninfected counterparts (22.0 days, IQR 0.0-92.0 days). Diagnostic delay was slightly shorter in PLWH (median 191.0 days, IQR 88.0-424.0 days) than in HIV-uninfected participants (median 204.0 days, IQR 61.0-350.0 days). Median time-to-treatment was 22.0 days (IQR 6.0-44.0 days) in PLWH, whereas it was 18.0 days (IQR 4.0-77.0 days) in HIV-uninfected patients (Table 1).

**TABLE 2:**
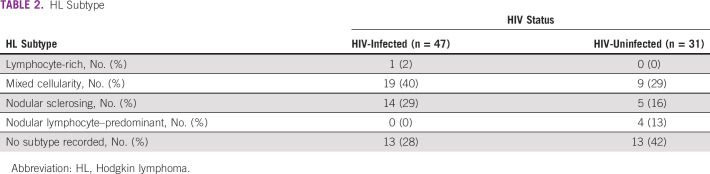
HL Subtype

### Treatment

Twenty-eight (60%) of the PLWH cohort received four to six cycles of ABVD, whereas 11 (35%) of the HIV-uninfected cohort received four to six cycles of ABVD. Seven (15%) of the PLWH group received regimens categorized as other or uncertain. Nine (29%) of HIV-uninfected subjects received an other or uncertain regimen. There was no record of chemotherapy for 11 (23%) PLWH, whereas 8 (32%) HIV-uninfected subjects did not have a record of chemotherapy. There was no statistically significant difference in chemotherapy regimen by HIV status (*P* = .20; Table 3).

**TABLE 3:**
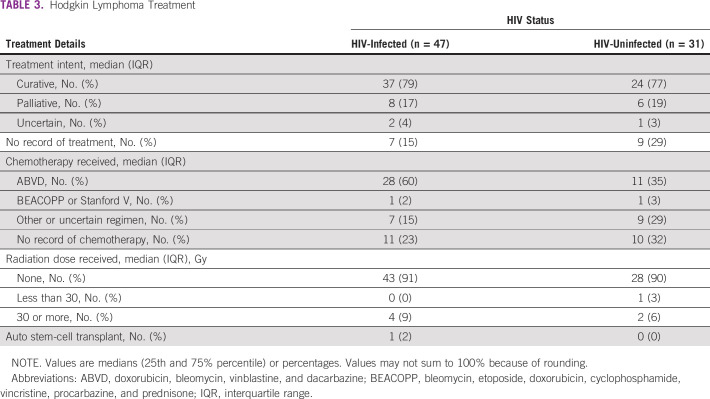
Hodgkin Lymphoma Treatment

### Survival

**FIG 1:**
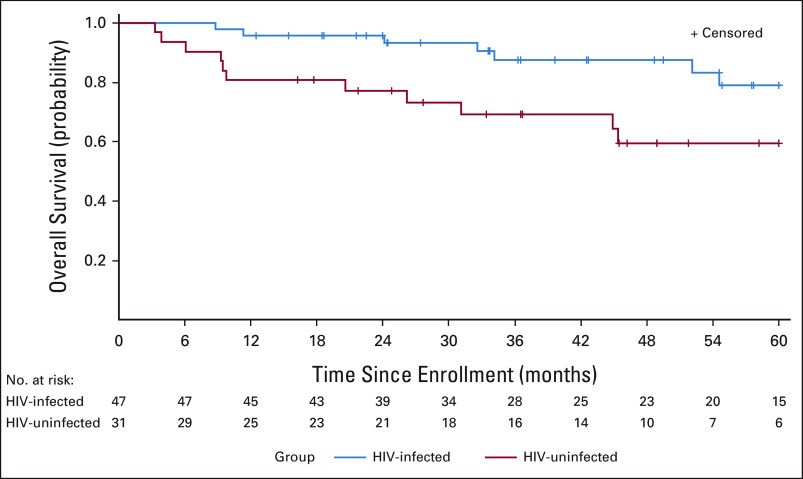
Estimated overall survival of Hodgkin lymphoma by HIV infection status using the Kaplan-Meier method. In this unadjusted analysis, patients with concurrent HIV infection experienced longer survival (log-rank *P* = .021). Shaded bands indicate 95% CI.

Median follow-up time among survivors was 51.4 months (IQR 33.8-60.0 months). No subjects were lost to follow-up. There were 18 total deaths in the study, seven PLWH and 11 without HIV. HL was reported as the primary cause of death in 16 participants, and complication of HL treatment in one patient (cause of death could not be obtained for one patient). Estimated 2-year survival for PLWH was 96% (95% CI, 93 to 99) and 74% (95% CI, 57 to 90) or HIV-uninfected patients. Overall survival, in unadjusted analyses, was lower among patients without HIV compared with PLWH (log-rank *P* = .021; Fig 1; Appendix Table A1). However, following adjustment for age, HL stage, and performance status, HIV infection was not significantly associated with survival in inverse probability–weighted Cox model (hazard ratio 0.43; 95% CI, 0.16 to 1.16; *P* = .094).

## DISCUSSION

In this prospective analysis of patients treated for HL in Botswana, we found that patients living with HIV had long-term outcomes at least as favorable as those without HIV. In unadjusted analysis, PLWH had superior survival. Following adjustment for stage, age, and performance, survival was not significantly different.

High utilization of ART, with consequent successful virologic suppression, and a health system with familiarity treating patients with HIV, could account for the lack of disparity in outcomes that has been seen in Europe.^18^ In many high-income countries, the HIV epidemic disproportionally affects marginalized communities,^20^ and differential health care access between HIV HL and non-HIV HL patients could contribute to disparity not seen in Botswana. Indeed, because of the stigma associated with an HIV diagnosis, we could also speculate differential psychosocial barriers to care in the HIV-positive populations of Botswana, Europe, and the United States. Although we did not identify large differences in time to treatment, longitudinal HIV care may facilitate access to diagnosis and therapy for HL among PLWH compared with patients without HIV.

Key biologic differences in HL between HIV-infected and HIV-uninfected patients have been identified. It has been suggested that HIV HL is more likely to be aggressive and present with B symptoms than non-HIV HL.^11^ Unfortunately, presence of B symptoms was not reliably recorded in our cases with missing information in 60% of enrolled participants. Studies have examined differential gene expression and identified EBV infection as a contributor to lymphomagenesis in almost all cases of HIV HL, relative to very few in non-HIV HL.^11-14^ One study suggested that HIV HL is derived from postgerminal center B cells,^15^ which could indicate a more differentiated lymphoma relative to non-HIV HL. Studies have also shown that ART affects the tumor microenvironment.^11^

These differences could confer a survival benefit, particularly the finding suggesting a more differentiated, postgerminal center B-cell phenotype in HIV HL.^15^ Botswana's high ART coverage could be affecting how HIV HL patients present in the country. Further molecular studies comparing HIV HL and non-HIV HL could illuminate possible improved outcomes in HIV-associated HL. This is particularly important in context of the aging HIV-infected population and the bimodal age incidence of HL and deceased survival in late-onset HL.^21-23^

The findings should be interpreted in the context of important study limitations. Treatment information regarding treatment intent and specific therapy was incomplete. This was more so the case in patients without HIV. It is possible that differences in curative versus palliative intent between HIV HL and non-HIV HL, as well as specific regimen, could have affected study findings.^25^ The sample size of 78 patients reflects both the rarity of HL and Botswana's small population. It is entirely possible that not all people with HL in Botswana presented for oncology care, whereas some might have obtained care outside of the country. Additionally, the timely diagnosis of HL remains a challenge in Botswana, given its similar presentation to disseminated infections such as tuberculosis.^24^ Some HL subtypes share histologic features with certain non-HLs and reactive conditions, and require immunohistochemistry to establish diagnosis with certainty. Resource limitations in the government health scheme meant we were not able to reliably obtain immunophenotyping information, or EBV -encoded RNA status on all tumors. Therefore, we were not able to consider HL subtype or EBV coinfection in our analysis, which could further illuminate the effect of HIV on presentation and survival of HL in high ART coverage settings. The pathologic reports generated through routine histology seemed to suggest PLWH were developing different HL subtypes to those without HL. HL subtype data were missing for 26 patients; so, this could not be investigated further. We also cannot exclude the possibility that our results could have been confounded by misdiagnoses, as external pathology review of HL and NHL cases in Botswana suggest.^26,27^

In conclusion, in this cohort receiving longstanding ART, patients with HL living with HIV experienced comparable survival to their HIV-uninfected counterparts. Molecular genetic studies comparing HL cases are indicated to explore whether differences in HIV HL may confer a clinical benefit.

## Figures and Tables

**TABLE A1:**
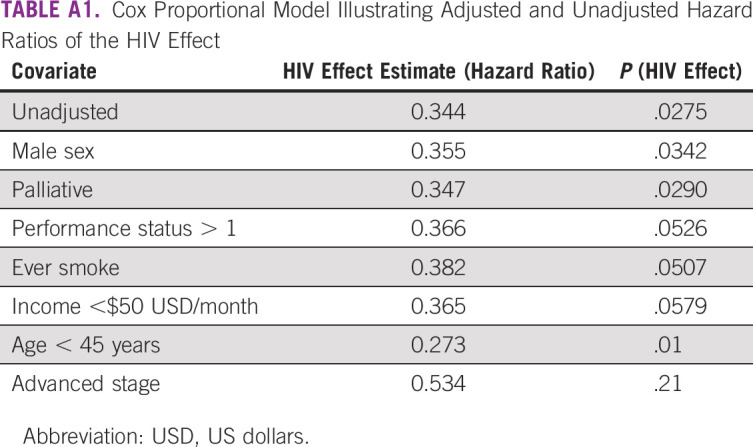
Cox Proportional Model Illustrating Adjusted and Unadjusted Hazard Ratios of the HIV Effect
